# Correction: Fairness and efficiency considerations in COVID-19 vaccine allocation strategies: A case study comparing front-line workers and 65–74 year olds in the United States

**DOI:** 10.1371/journal.pgph.0002785

**Published:** 2024-01-03

**Authors:** The PLOS Global Public Health Staff

There are errors in the Funding statement. The publisher apologizes for the errors. The correct Funding statement is as follows: ER and ML were supported by the Morris-Singer Fund. ML was supported, in part, by Award Number U01CA261277 from the US National Cancer Institute of the National Institutes of Health for this work. The content of this article is solely the responsibility of the authors and does not necessarily represent the official views of the Morris-Singer Fund or the National Institutes of Health. The funders had no role in study design, data collection and analysis, decision to publish, or preparation of the manuscript.

## References

[1] Rumpler E, Feldman JM, Bassett MT, Lipsitch M (2023) Fairness and efficiency considerations in COVID-19 vaccine allocation strategies: A case study comparing front-line workers and 65–74 year olds in the United States. PLOS Glob Public Health 3(2): e0001378. 10.1371/journal.pgph.0001378 36962865 PMC10021220

