# Supplementary figures and images for: Fairness and efficiency considerations in COVID-19 vaccine allocation strategies: A case study comparing front-line workers and 65–74 year olds in the United States
Source: PLOS Glob Public Health. 2023 Feb 6;3(2):e0001378. doi: 10.1371/journal.pgph.0001378 (PMC10021220; doi:10.1371/journal.pgph.0001378)

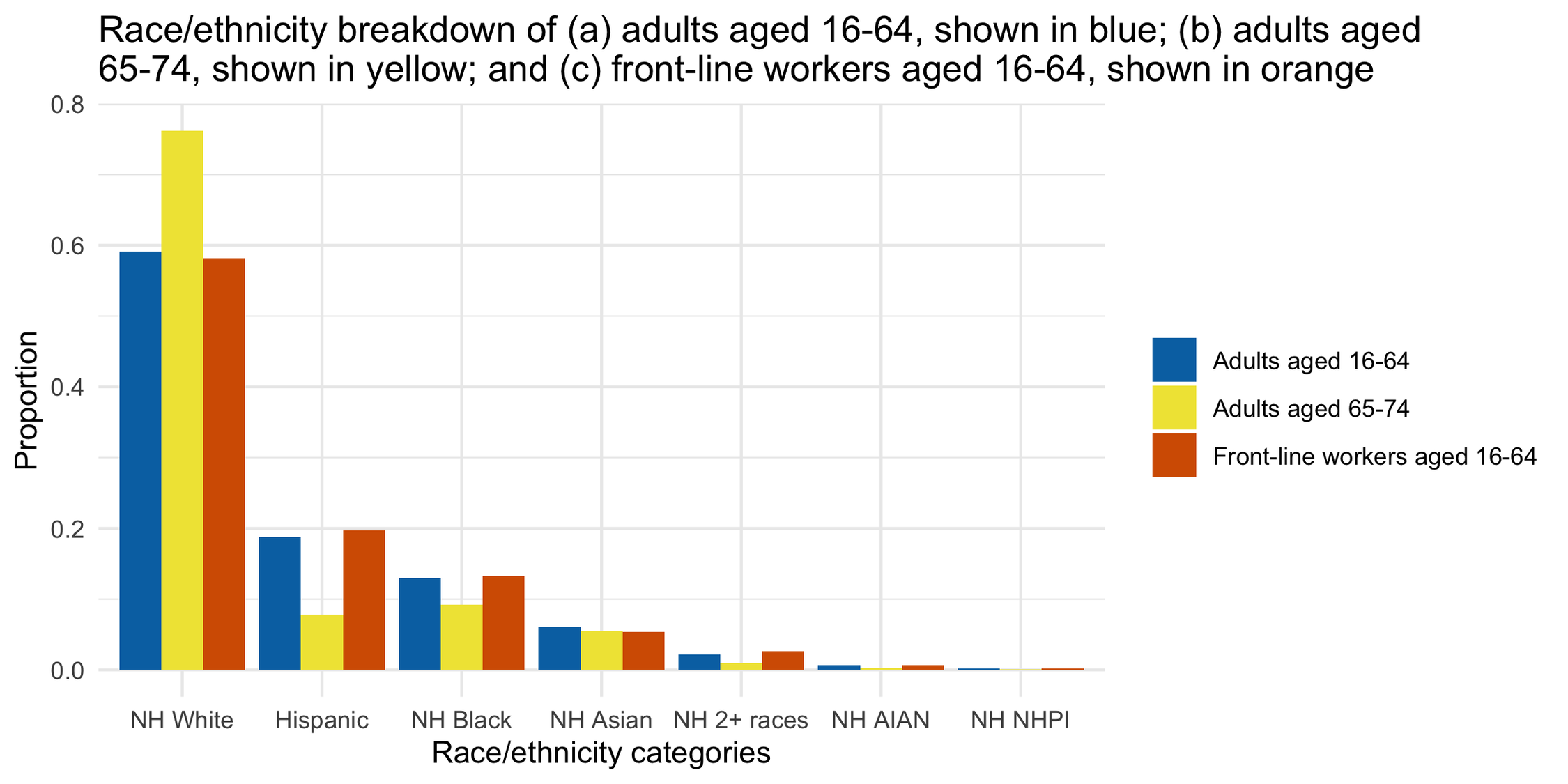

Supplement: S1 Fig — (TIF) [file pgph.0001378.s001.tif]

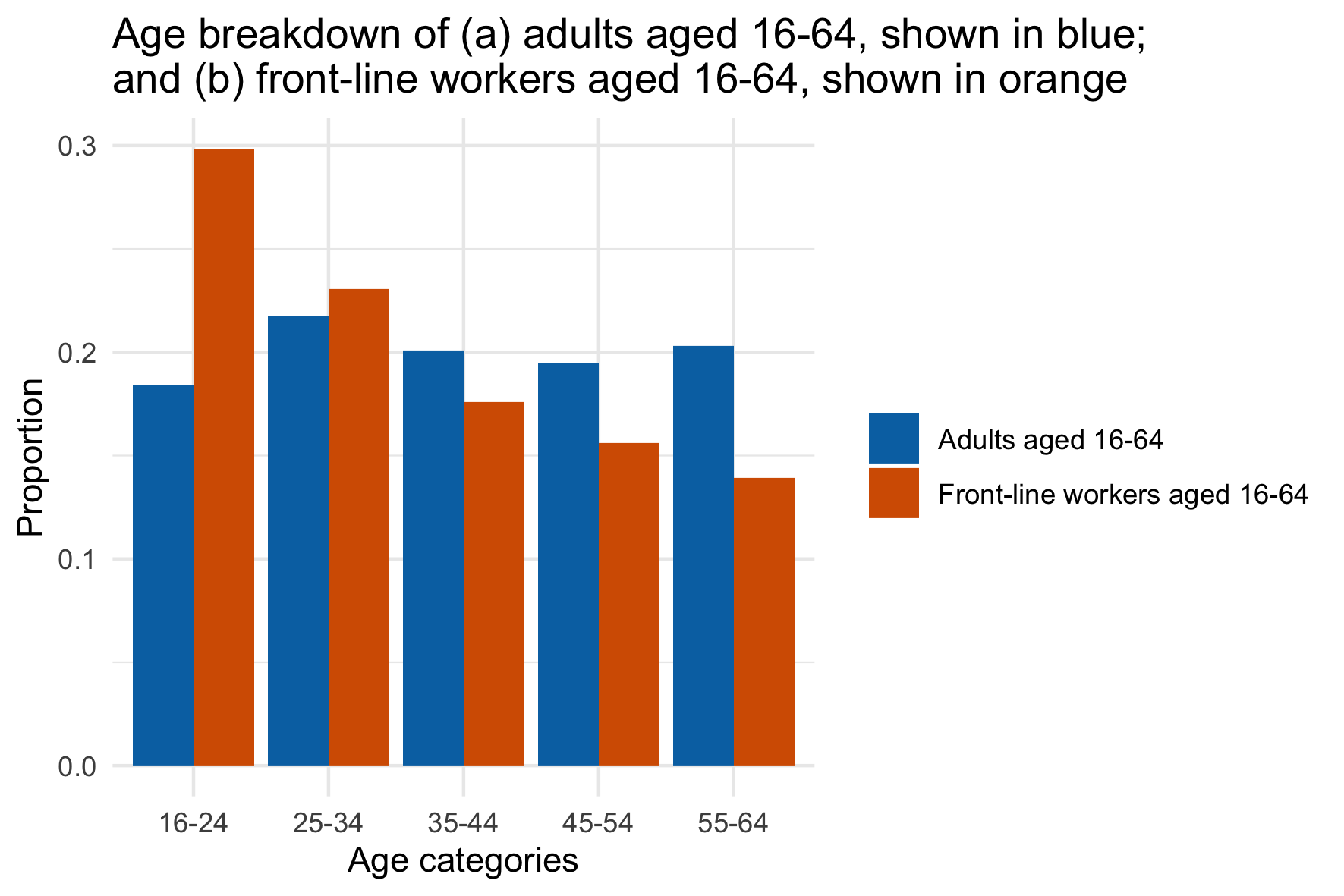

Supplement: S2 Fig — (TIF) [file pgph.0001378.s002.tif]

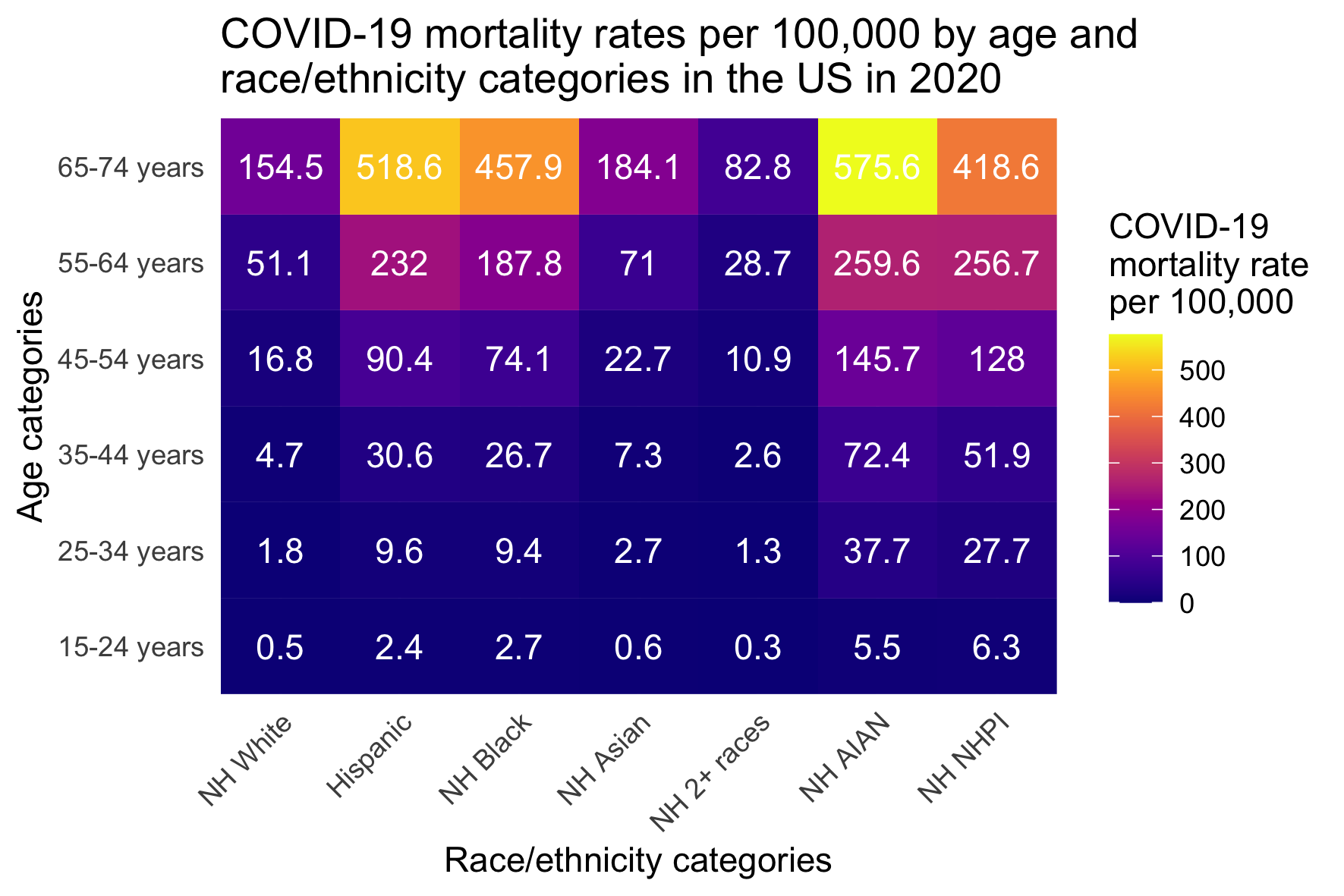

Supplement: S3 Fig — (TIF) [file pgph.0001378.s003.tif]
