# Supplementary material for: Fairness and efficiency considerations in COVID-19 vaccine allocation strategies: A case study comparing front-line workers and 65–74 year olds in the United States
Source: PLOS Glob Public Health. 2023 Feb 6;3(2):e0001378. doi: 10.1371/journal.pgph.0001378 (PMC10021220; doi:10.1371/journal.pgph.0001378)
Supplement: S2 Table — (DOCX) [file pgph.0001378.s005.docx]

**S2 Table. Expectation of life at age categories i in the USA in 2019 from the WHO GlobalHealth Observatory [18].**

| **Age category** | **SLE (years)** |
| --- | --- |
| 15-24 | 61.67 |
| 25-34 | 52.15 |
| 35-44 | 42.84 |
| 45-54 | 33.70 |
| 55-64 | 25.14 |
| 65-74 | 17.42 |
